# 1-methylnicotinamide modulates IL-10 secretion and voriconazole metabolism

**DOI:** 10.3389/fimmu.2025.1529660

**Published:** 2025-02-13

**Authors:** Xiaoyan Deng, Yuanqing Li, Lin Jiang, Xuqiu Xie, Xiaokang Wang

**Affiliations:** ^1^ Center of Community Health Service Management, Shenzhen Longhua District Central Hospital, Shenzhen, China; ^2^ Department of Pharmacy, Shenzhen Longhua District Central Hospital, Shenzhen, China; ^3^ Office of the Dean, Shenzhen Longhua District Central Hospital, Shenzhen, China

**Keywords:** 1-methylnicotinamide, kupffer cells, hepatocytes, voriconazole, CYP2C19, metabolism

## Abstract

**Background:**

Inflammatory diseases impair the hepatic metabolism of voriconazole (VRC). 1-Methylnicotinamide (1-MNA), a common final metabolite of nicotinamide in the liver, has demonstrated anti-inflammatory effects in recent studies. This study investigated the impact of 1-MNA on VRC metabolism in the liver.

**Method:**

Mice with a systemic inflammatory response induced by lipopolysaccharide (LPS) were intragastrically administered 1-MNA, and their VRC metabolic capacity was evaluated. Kupffer cells and primary hepatocytes were isolated, and flow cytometry along with molecular knockdown experiments were performed to explore the molecular mechanisms underlying improved drug metabolism. IL-10 knockout (IL-10^–/–^) mice were used to validate the role of IL-10 in enhancing hepatocyte VRC metabolism under inflammatory conditions.

**Results:**

1-MNA promoted M2 polarization of liver Kupffer cells, stimulated IL-10 secretion, upregulated CYP2C38 expression in primary hepatocytes, and enhanced VRC metabolism. The mechanism by which IL-10 upregulated CYP2C38 appears to involve the inhibition of the nuclear transcription factor NF-κB (p65) in hepatocytes.

**Conclusions:**

1-MNA regulated Kupffer cell polarization in an LPS-induced inflammatory environment, reduced the inflammatory inhibition of CYP2C38 expression in hepatocytes, and promoted VRC metabolism.

## 1 Introduction

The second-generation antifungal drug voriconazole (VRC; brand name VFEND^®^) is widely used for the prophylaxis and treatment of fungal infections caused by *Aspergillus* and *Candida* species (1). VRC is considered the first-line therapy for patients with immunodeficiency (2, 3). However, its nonlinear pharmacokinetics often result in drug accumulation, leading to various adverse effects, such as hepatotoxicity, rash, and optic nerve disorders (4–6). Inflammatory diseases can significantly affect the body’s ability to metabolize VRC (7, 8). Despite these findings, there is limited research on the specific mechanisms underlying impaired VRC metabolism in the context of inflammation.

Age, sex, and genetic variation in drug-metabolizing enzymes including *CYP2C19*, *FMO3*, and *CYP2C9* influence VRC metabolism (6, 9, 10). Both clinical and basic research indicate that changes in the clinical treatment environment, particularly changes in the internal milieu of hepatocytes, can significantly impact the liver’s capacity to metabolize VRC (11, 12). In a previous study by our research group, metabolomic analysis of people with different voriconazole metabolism abilities found that people with rapid voriconazole metabolizers had higher levels of 1-methylnicotinamide (1-MNA) in the peripheral blood, and were significantly negatively correlated with the pro-inflammatory factors interleukin (IL)-1β, tumor necrosis factor (TNF)-α, and the anti-inflammatory factor IL-10 is positively related. These levels are negatively correlated with pro-inflammatory factors, such as IL-1β and TNF-α, while showing a positive correlation with the anti-inflammatory factor IL-10.

1-MNA, the methylated amide of nicotinic acid, is produced by the enzymatic activity of nicotinamide N-methyltransferase and is primarily distributed in the liver (13). Previous studies have demonstrated the immunomodulatory properties of 1-MNA, including its ability to reduce reactive oxygen species (ROS) (14) and inhibit NLRP3 inflammasome activation (14). However, the immunomodulatory effects of 1-MNA on liver cells, particularly hepatocytes, remain unexplored.

Kupffer cells (KCs), the liver’s resident macrophages, play a central role in the hepatic immune response and inflammatory milieu (15). The M1 polarization of KCs is associated with increased secretion of pro-inflammatory cytokines, such as IL-1β and TNF-α (16), whereas M2 polarization (characterized by arginase-1 [Arg-1]) leads to elevated levels of the anti-inflammatory cytokine IL-10 (17, 18). The interplay between parenchymal and non-parenchymal liver cells is critical in driving inflammation and fibrosis, which may alter the liver’s drug metabolism capacity (19). Given that 1-MNA is highly expressed in rapid VRC metabolizers and inversely correlated with pro-inflammatory cytokines, we hypothesized that it may modulate inflammatory cytokine secretion by acting on KCs.

Lipopolysaccharide (LPS)-induced inflammatory conditions impair VRC metabolism (7). Previous research suggested that the LPS-induced NF-κB-p65 signaling pathway plays a role in downregulating CYP2C19, a key enzyme involved in VRC metabolism (20, 21). In addition, 1-MNA, an endogenous liver metabolite, influences the hepatic immune-inflammatory environment (22) and may enhance CYP2C19 expression. However, the precise mechanisms underlying this regulatory effect remain unclear.

We investigated the impact of 1-MNA on CYP2C19 expression and activity in hepatocytes within an LPS-induced inflammatory model. By elucidating the mechanism through which 1-MNA modulates VRC metabolism, we aim to provide novel insights into the interplay between liver inflammation and antifungal drug metabolism.

## 2 Materials and methods

### 2.1 Animals and treatment

C57BL/6J mice were obtained from the Laboratory Animal Center of Nanfang Hospital, Southern Medical University (Medical Experimental Animal Number: SCXK-2016-0041). 1-MNA was sourced from Sigma-Aldrich (St. Louis, MO, USA). C57BL/6J mice with an IL-10 knockout (IL-10^–/–^) background were purchased from Cyagen Biosciences (Suzhou, China). The IL-10^–/–^ mice were created by crossbreeding IL-10^–/–^ mice. 1-MNA was administered at a dose of 100 mg/kg body weight/day in drinking water as the vehicle. LPS was administered at a dose of 5 mg/kg body weight/day intraperitoneal injection. At the end of the study, 24 h after VRC administration, the mice were euthanized using an overdose of CO_2_. Liver samples were collected and subjected to immunofluorescence staining. All animal procedures and post-treatment were approved by the Animal Ethics Committee of Nanfang Hospital (Approval No. NFYY-2020–73).

### 2.2 VRC metabolic analysis

Blood samples were collected (into heparinized tubes) 4 h after a single 20 mg/kg dose of VRC. Plasma was separated by centrifugation at 13,000 rpm for 10 min, and the supernatant was collected. Additional plasma samples were centrifuged at 3,500 rpm and 4°C for 15 min. The supernatant was stored at –80°C until analysis. VRC metabolic analysis followed the methodology described in a previous study (20). The concentrations of VRC and its N-oxide derivative were measured using high-performance liquid chromatography coupled with tandem mass spectrometry (HPLC-MS/MS). The transparent supernatant was evaporated under nitrogen gas, redissolved in 100 µL acetonitrile with 0.1% formic acid (40/60, v/v), and analyzed through HPLC using standard solutions and quality control samples. Data were analyzed using PK Solver software. The VRC metabolic ratio was calculated using the following formula:


$$\frac{\text{C }\left(\text{Voriconazole N }–\text{ oxide}\right)}{\text{C }\left(\text{Voriconazole}\right)}\;\times \;100\%$$

### 2.3 Tissue preparation and cytokine detection

Livers of mice were processed through a 70 µm cell strainer (BD Biosciences, San Jose, CA, USA). Mononuclear cells were washed with phosphate-buffered saline (PBS), minced into small pieces, and resuspended in 40% Percoll (Sigma-Aldrich). The cell suspension was carefully layered onto 70% Percoll and centrifuged at 400 × g for 20 min. For hepatocyte or KC isolation, livers of mice were perfused with collagenase as previously described.

For enzyme-linked immunosorbent assay (ELISA) testing, reagents were prepared and samples were diluted at a 1:2 ratio. IL levels were measured using a standard curve. Liver levels of cytokines were quantified using ELISA kits (R&D Systems, Minneapolis, MN, USA) following the manufacturer’s instructions. Serum and supernatant concentrations of Arg-1, IL-6, IL-10, TNF-α, IL-17A, and IL-1β were measured. Cytokine levels are expressed as mass per milligram of tissue.

### 2.4 Preparation of microsomes from the livers of mice

Liver microsomes were prepared as described in a previous study (23). Freshly collected Livers of mice were homogenized in ice-cold normal saline at a 1:3 (m/V) ratio to create a liver homogenate. The tissue was homogenized in a buffer containing sucrose and a protease inhibitor cocktail (1 mL per 400 mg tissue). The homogenate was centrifuged twice at 10,800 × g for 15 min to remove cell debris. Then the supernatant was centrifuged at 800 × g for 20 min to isolate pink translucent microsomal pellets. All procedures were performed at 4°C. The microsomal pellet was resuspended in a storage buffer and stored at –80°C until use.

The liver microsome incubation system consisted of 5 mL microsomes (20 mg/mL), 2 mL VRC (1 mM), 20 mL NADPH (1.3 mM), and 0.05 mM Tris/HCl buffer (pH 7.4), up to a total volume of 200 mL. The negative control group, excluding liver microsomes, included 2 mL 1 mM VRC, 20 mL 1.3 mM NADPH, and 0.05 mM Tris/HCl buffer at pH 7.4, also totaling 200 mL.

### 2.5 Cell culture and treatments

Cells were cultured in DMEM supplemented with 10% fetal bovine serum, 100 U/mL penicillin, and 100 µg/mL streptomycin at 37°C in a 5% CO_2_ atmosphere, with treatments involving small interfering RNA (siRNA) oligonucleotides or control siRNA. The medium was changed every 3 days until the cells reached confluence. Hepatocytes were incubated for 30 min with or without LPS or IL-10. For mechanism studies, cells were harvested at different time points for polymerase chain reaction or immunoblot analysis. Each assay was repeated at least three times.

### 2.6 Cell counting kit-8 assay

Cell viability was assessed using a CCK8 assay (Boster, Wuhan, China). Cells were rinsed with PBS. A specified quantity of cells (200 μL or 1 × 10^4^) was centrifuged, resuspended in fresh medium, and 10 µL CCK8 reagent was added to each well of a 96-well plate. The plates were incubated at 37°C for 90 min. The control well contained no cells but included the medium and CCK8 reagent. Absorbance at 490 nm was measured after 48 h using a Biotek ELISA plate reader (Winooski, VT, USA). Cell viability was calculated using a formula from a previous study (20):


$$\frac{\left[\text{OD}\;(\text{Sample})\;-\;\text{OD}(\text{Blank})\right]}{\left[\text{OD}\;(\text{Control})\;-\;\text{OD}(\text{Blank})\right]}\;\times \;100\%$$

### 2.7 Quantitative reverse transcription-polymerase chain reaction

qRT-PCR was used to analyze the expressions of *Cyp2c38*, *Cyp2c29*, *Cyp3a11*, and genes encoding specific inflammatory cytokines and transcription factors (20). Samples were homogenized for mRNA extraction using TRIzol reagent (Invitrogen). mRNA concentration was measured with an ABI Prism 7900 Sequence Detection System (Applied Biosystems, Foster City, CA, USA). Highly homologous and comparable product profiles between human *Cyp2c19*, *Cyp2c9*, and *Cyp3a4*, and mouse *Cyp2c* isoforms *Cyp2c38*, *Cyp2c29*, *Cyp3a11*, respectively, have been established (24, 25). **Supplementary Table S1** provides the primer sequences for the target genes and the internal control gene, glyceraldehyde 3-phosphate dehydrogenase (GAPDH). The 2^–ΔΔCt^ method was used to quantify fold changes in relative gene expression, normalized to GAPDH, based on three replicates.

### 2.8 siRNA-mediated IL-10 gene knockdown

IL-10 selective siRNA oligonucleotides (20 nM) were obtained from Santa Cruz Biotechnology (catalog number: sc-39634). Cells were transfected with IL-10 siRNA using Lipofectamine™ 3000 (Thermo Fisher Scientific, Waltham, MA, USA) according to the manufacturer’s instructions. Then they were cultured to 85% confluence. Control siRNA sequences (catalog number: sc-37007; Santa Cruz) were transfected into cells using the same protocol. The cells were serum-starved for 12 h prior to transfection with 20 nM siRNAs. Transfected cells underwent either protein extraction or immunofluorescence staining.

### 2.9 Flow cytometry

KCs were efficiently isolated from liver samples using the three-step method (26). The cells were stained with surface antibodies according to the manufacturer’s guidelines. Each group of KCs was fixed in 4% buffered formaldehyde for 10 min, permeabilized with 0.2% Triton X-100 for 30 min, and blocked with 1% bovine serum albumin (BSA) for 30 min, all at room temperature. Then the KCs were rinsed with PBS at room temperature for 5 min. They were incubated overnight at 4°C, shielded from light, with primary antibodies targeting F4/80, CD206, and iNOS, each at a dilution of 1:200. The fluorescent antibodies are listed in **Supplementary Table S2**. Next, the KCs were incubated with a secondary antibody (1:1,000) at room temperature for 1 h, shielded from light. Finally, they were incubated with DAPI at 37°C for 30 min, protected from light, and kept at room temperature for 10 min. After sealing with a fluorescent quenching agent, KC staining was examined via fluorescence microscopy and analyzed using the FlowJo software (27). Cells exhibiting an M1 phenotype were characterized by F4/80^+^ iNOS^+^ CD206^–^ markers, while the M2 phenotype was indicated by F4/80^+^ iNOS^–^ CD206^+^ markers.

### 2.10 Chromatin immunoprecipitation

ChIP assays were performed using a SimpleChIP Plus Enzymatic ChIP kit as previously described (20, 28). Livers of mice were preserved using formaldehyde. Following sample lysis, chromatin was sheared and subjected to immunoprecipitation with either anti-p65 or normal IgG. DNA fragments were purified and quantified using qPCR with specific primers (**Supplementary Table S1**).

### 2.11 Immunofluorescence staining

Each group of KCs and hepatocytes was fixed in 4% buffered formaldehyde for 10 min, permeabilized with 0.2% Triton X-100 for 30 min, and blocked with 1% BSA, all at room temperature. KCs or hepatocytes were washed with PBS at room temperature for 5 min. The KCs were incubated overnight at 4°C, shielded from light, with primary antibodies targeting iNOS and IL-10, both at a dilution of 1:200. The hepatocytes were incubated with primary antibodies against p65 (1:200). Both sample types were washed and kept in the dark at room temperature for 5 min. They were incubated with a secondary antibody (1:1,000) at room temperature for 1 h, protected from light and subsequently washed and shielded from light at room temperature for 5 min. Finally, they were incubated with DAPI at 37°C for 30 min, protected from light. They were stained and observed using fluorescence microscopy after sealing with a fluorescent quenching agent.

### 2.12 Statistical analyses

Experimental data are reported as means and standard deviations (SDs). GraphPad Prism 8.0 was used for data analysis. The Student’s *t*-test was used to assess differences between two groups, while the Kruskal-Wallis test followed by the Dunn’s *post hoc* test were used for comparisons among multiple groups. Linear regression analysis was performed using the loss minimization function in R 3.5.3 (R Core Team, Vienna, Austria). Other statistical methods are reported accordingly. Results with *P* < 0.05 were considered statistically significant.

## 3 Results

### 3.1 1-MNA inhibits the inflammatory response in the liver and upregulates the expression of the VRC-metabolizing enzyme CYP2C38

**Figure 1:**
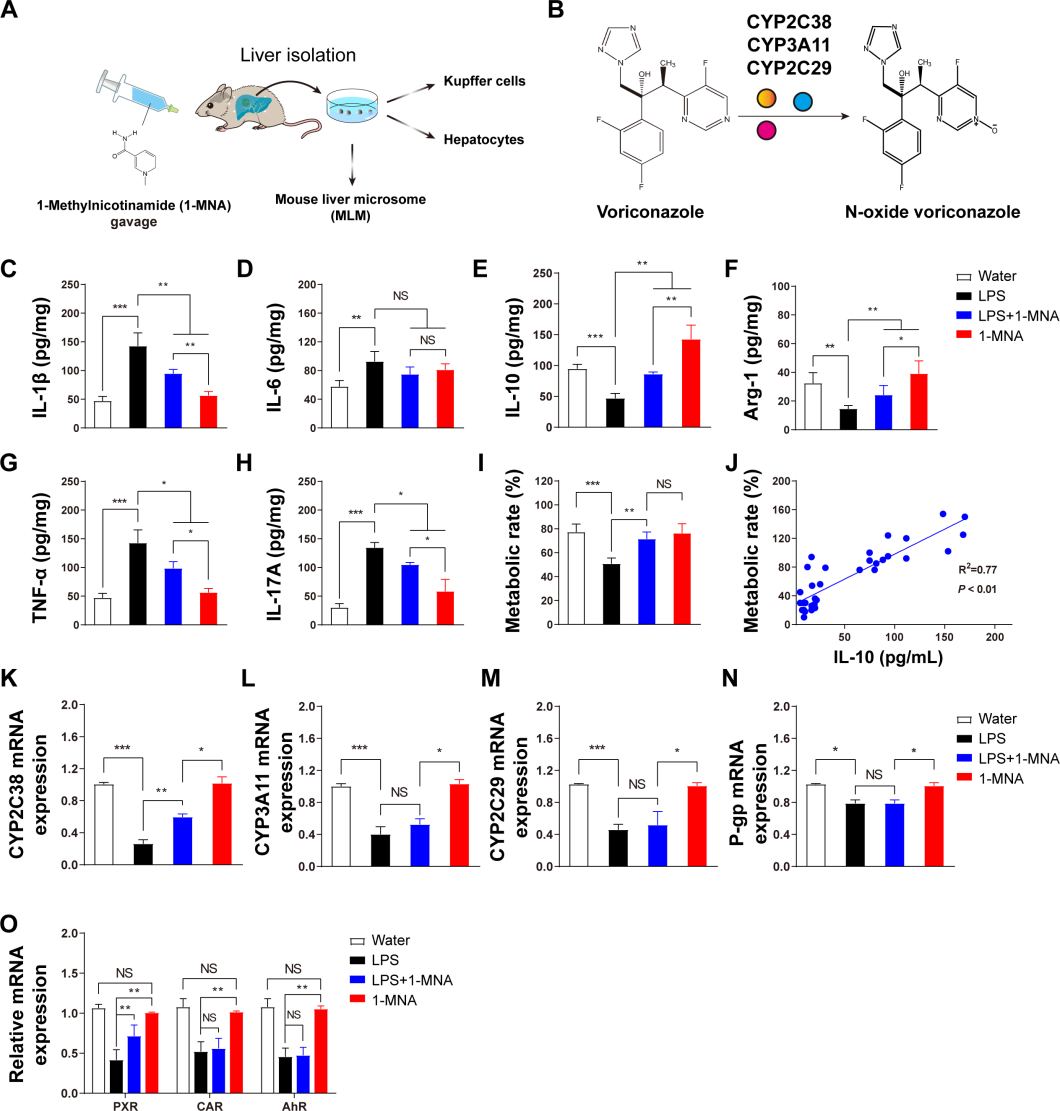
The inflammatory response in the liver is reduced and voriconazole metabolism is improved after 1-MNA administration. **(A)** Flowchart depicting the experimental verification of the regulation of voriconazole metabolism by 1-MNA using an LPS-induced animal inflammation model. The chemical structure of 1-MNA in the intragastric solution is shown. 1-MNA was administered at a dose of 100 mg/kg body weight/day in drinking water as the vehicle. **(B)** Schematic diagram of voriconazole metabolism, highlighting its conversion into the non-hepatotoxic nitrogen oxide compound (N-oxide VRC) by drug-metabolizing enzymes (CYP2C38, CYP3A11, or CYP2C29). **(C–H)** Quantitative analysis of inflammatory factors in liver tissue: IL-1β **(C)**, IL-6 **(D)**, IL-10 **(E)**, Arg-1 **(F)**, TNF-α **(G)**, and IL-17A **(H)**. **(I)** Comparison of voriconazole metabolism rates among groups, determined after microsomes isolated from the livers of mice were incubated with VRC for 4 h *in vitro*. **(J)** Correlation analysis between hepatic IL-10 levels and voriconazole metabolism rates, with R^2^ and *p*-values shown (n = 34). **(K–N)** Gene expression analysis of voriconazole-related drug-metabolizing enzymes and transporters: CYP2C38 **(K)**, CYP3A11 **(L)**, CYP2C29 **(M)**, and P-glycoprotein **(N)**. **(O)** Analysis of key transcription factor genes involved in the regulation of drug-metabolizing enzymes in primary hepatocytes. *p < 0.05, **p < 0.01, ***p < 0.001; NS: no significance.

The systemic inflammatory response model induced by LPS suppresses the expression of hepatic drug-metabolizing enzymes. In this study, we investigated the impact of 1-MNA on the inflammatory response in the liver and its regulation of drug-metabolizing enzymes, particularly in relation to VRC metabolism. Liver microsomes, hepatocytes, and KCs were isolated from liver tissues following intragastric administration of 1-MNA (**Figure 1A**). Given that VRC metabolism is influenced by three key drug-metabolizing enzymes, CYP2C38, CYP2C29, and CYP3A11, we sought to elucidate the effects of 1-MNA on these enzymes and the potential mechanisms involved (**Figure 1B**).

To assess the inflammatory response, we quantitatively analyzed the expression of four pro-inflammatory cytokines (IL-1β, IL-6, TNF-α, and IL-17A) and one anti-inflammatory cytokine (IL-10) in the liver tissues across experimental groups. The LPS-induced model group showed a marked increase in pro-inflammatory cytokine expression (**Figures 1C, D, G, H**) and a significant reduction in anti-inflammatory cytokines (IL-10) and the M2 macrophage marker Arg-1 (**Figures 1E, F**). Following 1-MNA administration, there was a significant reduction in pro-inflammatory cytokines and an increase in anti-inflammatory markers in the liver tissues of both the LPS model and normal rats (**Figures 1C–H**).

Furthermore, we analyzed the relationship between inflammatory cytokine levels and the metabolic rate of VRC. An increase in IL-10 levels was positively correlated with an enhanced metabolic rate of VRC in the liver (**Figures 1I, J**). These findings indicate that IL-10 may facilitate improved VRC metabolism in an inflammatory hepatic environment, although the precise mechanism underlying this effect remains unclear.

Gene expression analysis of the drug-metabolizing enzymes revealed that 1-MNA significantly upregulated the expression of *Cyp2c38* in an LPS-induced inflammatory environment, while having no observable effect on *Cyp2c29*, *Cyp3a11*, or *P-glycoprotein* gene expression. In addition, we assessed the expression of nuclear transcription factors involved in the regulation of drug-metabolizing enzymes. The expression of the pregnane X receptor (PXR) was significantly increased following 1-MNA administration, suggesting that 1-MNA may enhance *Cyp2c38* gene expression by upregulating PXR expression under inflammatory conditions.

### 3.2 1-MNA promotes IL-10 secretion by regulating KC polarization

**Figure 2:**
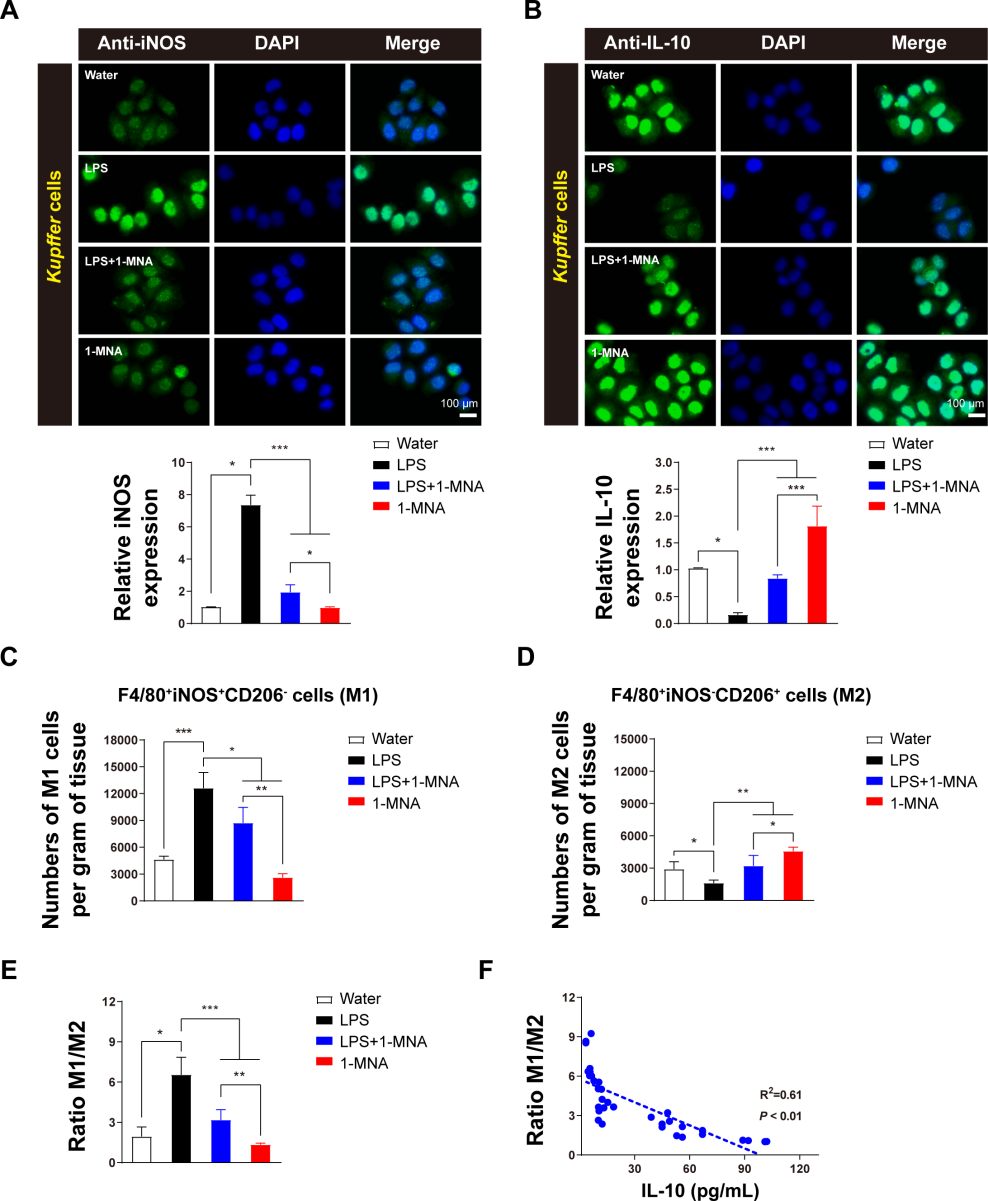
1-MNA promotes M1 to M2 polarization of KCs. **(A)** Cultured KCs with iNOS expression. Top: Representative immunofluorescent staining of KCs using anti-iNOS (green) antibodies. Scale bar: 100 µm. Bottom: Quantification of iNOS staining in KCs (n = 3). **(B)** Cultured KCs with IL-10 expression. Top: Representative immunofluorescent staining of KCs using anti-IL-10 (green) antibodies. Scale bar: 100 µm. Bottom: Quantification of IL-10 staining in KCs (n = 3). **(C, D)** Analysis of M1 **(C)** and M2 **(D)** cell counts in liver tissue across animal groups. Macrophages were classified as M1 (F4/80^+^, iNOS^+^, CD206)^–^ and M2 (F4/80^+^, iNOS^-^, CD206^+^). **(E)** Analysis of the M1/M2 ratio in liver tissue across animal groups. **(F)** Correlation between IL-10 production and macrophage polarization. R^2^ and *P*-values for the regression model are shown (n = 34). Values are expressed as means ± SD from three independent experiments. *p < 0.05, **p < 0.01, ***p < 0.001, compared to the paired group. Statistical comparisons between groups were performed using the Kruskal-Wallis test, followed by Dunn’s *post hoc* test to detect differences among groups.

To investigate the anti-inflammatory effects of 1-MNA and its role in upregulating *CYP2C38* expression, KCs were isolated from liver tissue, cultured, and analyzed *in vitro*. Immunofluorescence analysis revealed that 1-MNA significantly increased the expression of the M1 macrophage marker protein iNOS (**Figure 2A**) and the M2 macrophage marker protein IL-10 (**Figure 2B**).

Flow cytometric analysis of liver tissue showed that, compared to a water-treated group, the number of F4/80^+^iNOS^+^CD206^–^ cells (M1 macrophages) induced by LPS was significantly higher. However, administration of 1-MNA resulted in a substantial reduction in these pro-inflammatory cells compared to the LPS group (**Figure 2C**). Conversely, the number of F4/80^+^iNOS^-^CD206^+^ cells (M2 macrophages) was significantly lower in the LPS-induced group than in controls but increased markedly following 1-MNA administration (**Figure 2D**).

Comparative analysis of KC polarization types revealed that the M1/M2 ratio in LPS-induced inflammatory rats was significantly reduced following 1-MNA administration. This was accompanied by a significant increase in IL-10 levels in liver tissue (**Figure 2E**). Correlation analysis further demonstrated a significant negative association between the M1/M2 ratio and IL-10 levels, with R^2^ = 0.61 (**Figure 2F**). These findings suggest that 1-MNA mitigates the pro-inflammatory response by promoting KC polarization toward the anti-inflammatory M2 phenotype.

### 3.3 1-MNA upregulates CYP2C38 expression in hepatocytes via IL-10 secretion

**Figure 3:**
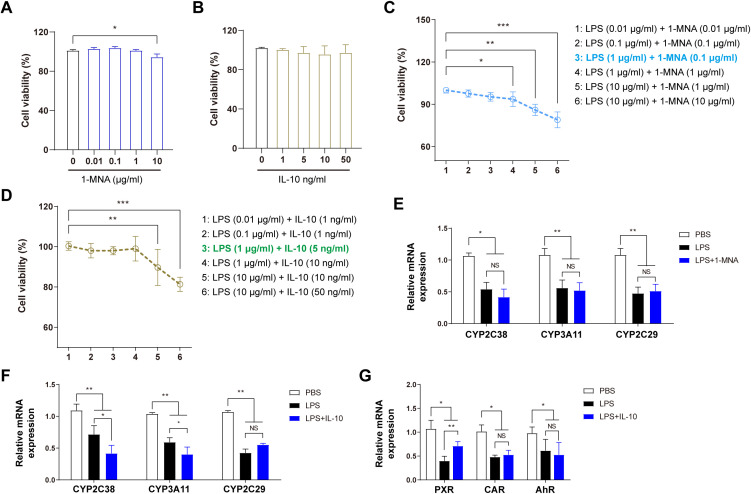
1-MNA indirectly upregulates CYP2C38 expression by promoting IL-10 secretion. **(A)** Effect of 1-MNA on primary hepatocyte activity at different concentrations. **(B)** Effect of IL-10 on primary hepatocyte activity at different concentrations. **(C)** Optimization of 1-MNA and LPS concentrations based on primary hepatocyte activity. **(D)** Optimization of IL-10 and LPS concentrations based on primary hepatocyte activity. **(E)** Analysis of drug-metabolizing enzyme gene expression in primary hepatocytes following 1-MNA administration. **(F)** Analysis of drug-metabolizing enzyme gene expression in primary hepatocytes following IL-10 administration. **(G)** Analysis of drug-metabolizing enzyme nuclear transcription factor gene expression in primary hepatocytes following 1-MNA administration. *p < 0.05, **p < 0.01, ***p < 0.001, compared to the paired group. NS, no significance.

Mechanistic experiments were conducted on isolated primary cells to elucidate how 1-MNA and IL-10 enhance hepatic enzyme activity, correlated with *in vivo* outcomes. The CCK8 assay was used to determine the optimal concentrations of 1-MNA, IL-10, and LPS for direct administration to liver cells. As shown in **Figure 3**, 1-MNA concentrations exceeding 10 μmol/L significantly reduced hepatocyte viability. IL-10 did not significantly affect liver cell viability within the range of 1–50 ng/mL. The effects of combining LPS with either 1-MNA or IL-10 on cell viability were also assessed. In combination with LPS (1 μg/mL), 1-MNA (0.1 μg/mL) did not significantly affect cell viability (*P* > 0.05), IL-10 (10 μg/mL) significantly reduced cell viability (P < 0.05), and IL-10 (5 μg/mL) had no effect on hepatocyte viability.

To create an efficient inflammatory cell model without compromising cell viability, the *in vitro* LPS concentration was established at 1 μg/mL. These findings guided further exploration of the effects of LPS combined with 1-MNA or IL-10 on CYP450 activity and expression. The optimal concentrations for the *in vitro* model were determined as 1 μg/mL LPS (inflammatory stimulus), 0.1 μg/mL 1-MNA, and 5 ng/mL IL-10 (**Figure 3D**).

Following the combined administration of 1-MNA and LPS to hepatocytes, the expression of CYP450 enzymes (CYP2C38, CYP3A11, and CYP2C29) did not significantly differ from hepatocytes treated with LPS alone (**Figure 3E**), indicating that 1-MNA does not directly upregulate CYP450 enzyme expression. Conversely, the combined administration of IL-10 with LPS resulted in a significant increase in the expressions of CYP2C19 and CYP3A4 (**Figure 3F**) and upregulation of PXR transcriptional expression under LPS-induced pro-inflammatory conditions (**Figure 3G**).

### 3.4 IL-10 upregulates PXR expression by inhibiting p65 transcription in LPS-treated hepatocytes

**Figure 4:**
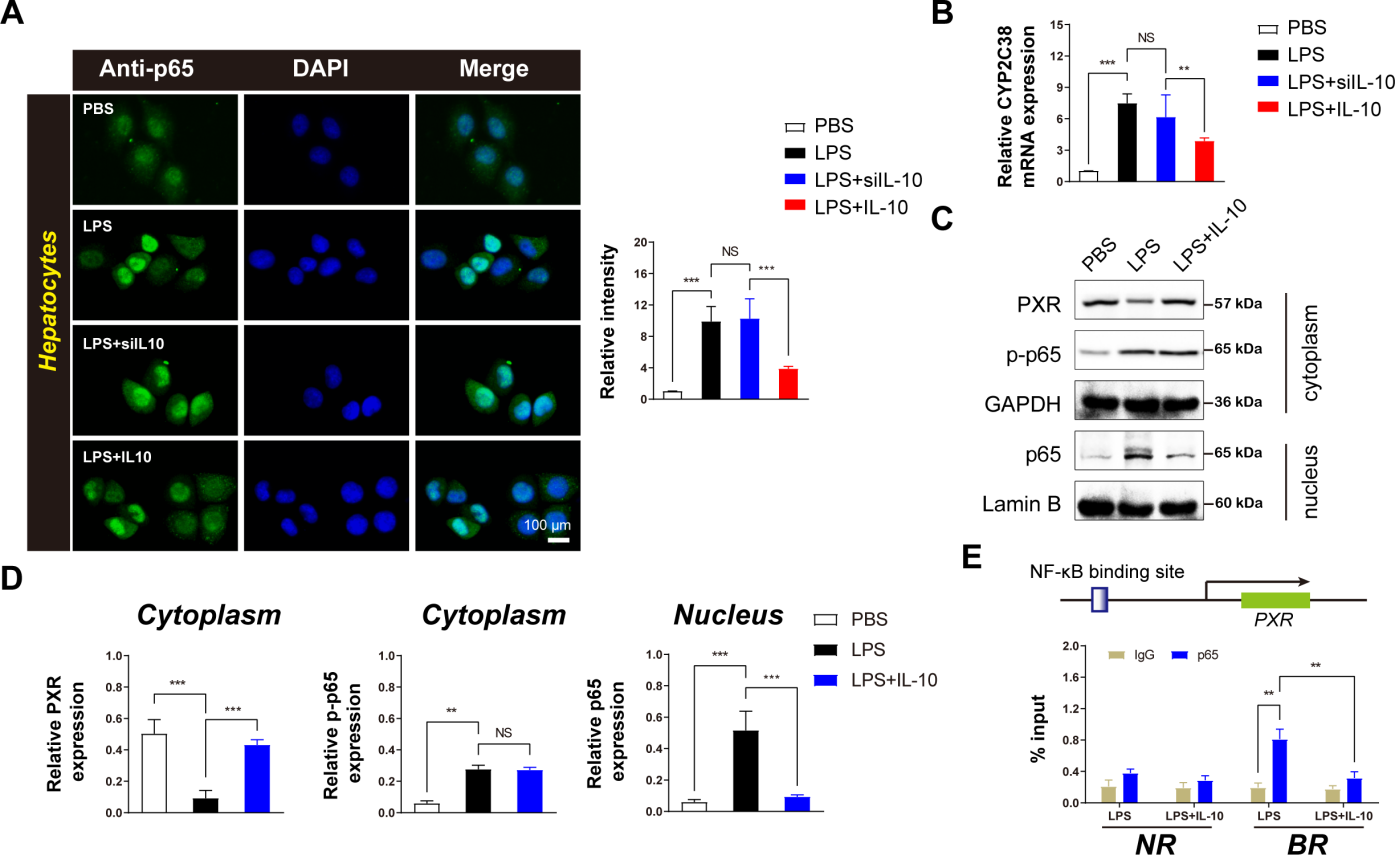
IL-10 inhibits the inflammatory transcription factor p65 in the nuclei of hepatocytes and upregulates the expression of CYP2C38 metabolic enzyme. **(A)** Left: Immunofluorescence analysis of effect of IL-10 on p65 expression in hepatocytes (scale bar = 100 μm). Right: Densitometric analysis of protein expression. **(B)** Analysis of *CYP2C38* gene expression in hepatocytes across groups. **(C)** Western blotting analysis of p65 and PXR protein expression in hepatocytes. **(D)** Quantification of target protein levels in the cytoplasm and nucleus based on panel **(C)**. **(E)** ChIP assay of p65 enrichment in the PXR promoter binding region in the nucleus after IL-10 treatment. BR: binding region; NR: non-binding region. Data are expressed as means ± standard deviation. **P < 0.01, ***P < 0.001 compared to the paired group; NS: no significance.

NF-κB-p65 in hepatocyte nuclei significantly inhibits the transcription of *CYP2C19* (20). To further explore the effect of IL-10 on p65 expression in hepatocytes, immunofluorescence experiments were conducted on primary hepatocytes. IL-10 administration significantly reduced p65 expression in hepatocytes, an effect that was abolished when IL-10 expression was silenced (**Figure 4A**). These findings suggest that IL-10 inhibits p65 expression in LPS-induced inflammatory hepatocytes.

Furthermore, IL-10 administration upregulated *CYP2C38* expression in inflammatory hepatocytes (**Figure 4B**), indicating a direct regulatory role of IL-10 in enhancing *CYP2C38* expression under inflammatory conditions.

To elucidate the underlying mechanism, Western blotting analysis was performed to assess p65 expression in the cytoplasm and nucleus. IL-10 administration inhibited nuclear p65 expression without affecting cytoplasmic levels (**Figure 4C**). In addition, cytoplasmic PXR expression was significantly increased (**Figures 4C, D**). These findings demonstrate that IL-10 specifically suppresses nuclear p65 activity in inflammatory hepatocytes.

ChIP analysis was used to further investigate the regulatory role of NF-κB on PXR transcription *in vivo* (**Figure 4E**). The results demonstrated that activated NF-κB-p65 was recruited to PXR-binding sites in hepatocytes, suppressing its expression. However, IL-10 administration significantly reduced p65 recruitment at the PXR-binding site, while no significant differences were observed in p65 accumulation at distal non-binding regions (NRs) (**Figure 4E**).

These results suggest that activated NF-κB specifically inhibits PXR transcription in hepatocytes and that IL-10 administration reverses this effect, highlighting the critical regulatory role of IL-10 in modulating the NF-κB/PXR pathway in inflammatory hepatocytes.

### 3.5 IL-10 reduces VRC accumulation by regulating the p65-PXR signaling pathway *in vivo*

**Figure 5:**
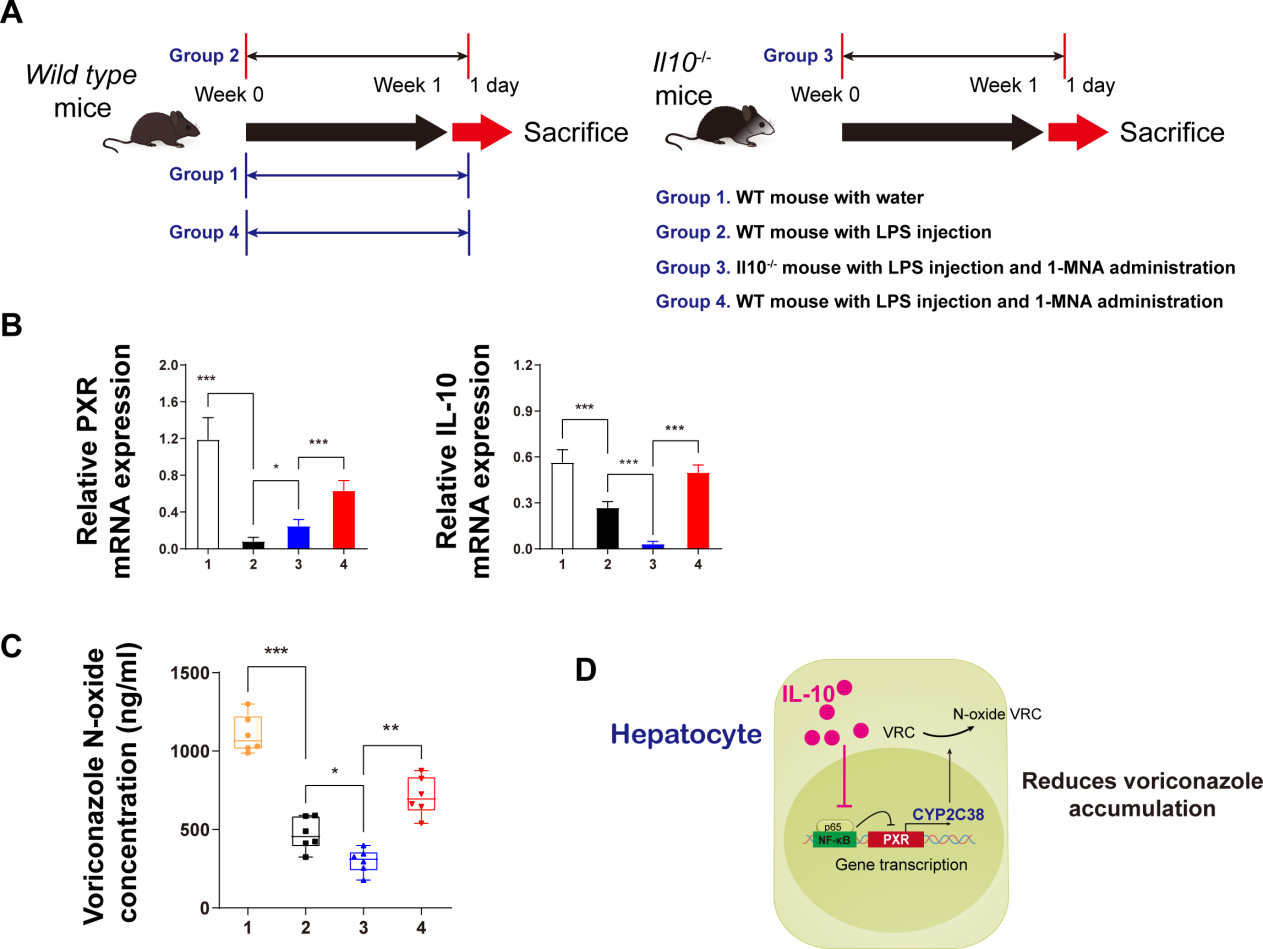
IL-10 promotes voriconazole metabolism in hepatocytes and reduces voriconazole accumulation in the liver. **(A)** Schematic diagram of the *in vivo* animal experiment flow. **(B)** mRNA expression analysis of IL-10 and PXR protein levels in primary hepatocytes from animals in each group. **(C)** After incubating isolated liver microsomes with voriconazole for 4 h, the concentration of voriconazole N-oxides in each group was quantitatively analyzed. **(D)** Schematic diagram illustrating the mechanism by which IL-10 reduces voriconazole accumulation. Data are expressed as means ± standard deviation. *P < 0.05, **P < 0.01, ***P < 0.001 compared to the paired group.

*In vivo* validation of the findings in animal models is crucial for understanding potential drug interactions. To confirm the results, IL-10 gene knockdown (IL-10^–/–^) mice were used for further investigation. The experimental design included four groups, with Group 3 involving IL-10^–/–^ mice and a 1-week combined treatment of LPS and 1-MNA. On the first day following the week-long treatment, liver microsomes were isolated for pharmacokinetic testing to assess the effects of different treatments on VRC metabolism (**Figure 5A**).

As anticipated from the *in vitro* data (**Figures 5B**), IL-10 knockdown *in vivo* led to a reduction in PXR expression in the cytoplasm, indicating that IL-10 upregulates PXR transcriptional expression. The mechanism likely involves IL-10 inhibiting the nuclear transcription of p65.

Regarding VRC metabolism, we measured VRC nitrogen oxide levels 4 h after administering the drug to isolated liver microsomes (**Figure 5C**). In IL-10 knockdown mice, the concentration of VRC nitrogen oxides was significantly lower than in the other three groups. By contrast, LPS-treated hepatitis mice administered 1-MNA exhibited significantly higher nitrogen oxide levels compared to both the LPS and IL-10 knockdown groups.

Based on these findings, a mechanistic model was proposed: IL-10 upregulates the expression of *CYP2C38*, which metabolizes VRC, by reducing the inhibitory effects of nuclear p65 on PXR transcription. This ultimately leads to reduced accumulation of the parent compound, VRC, in the liver (**Figure 5D**).

## 4 Discussion

The prevention and treatment of VRC-induced hepatotoxicity remain critical clinical challenges, with limited research focusing on the use of endogenous substances to mitigate these adverse effects. Identifying and developing substances to predict or intervene in VRC-induced hepatotoxicity is of significant clinical importance. This study demonstrates that the endogenous liver substance 1-MNA modulates liver immune function by promoting M2 polarization of KCs, upregulating IL-10 expression, inhibiting p65 expression in hepatocytes, and enhancing the expression of the enzyme responsible for VRC metabolism.

Our findings provide novel evidence that 1-MNA administration to mice results in enhanced VRC metabolism and increased IL-10 secretion in the liver, along with significant upregulation of CYP2C38 expression. While CYP2C19 is the primary metabolic enzyme for VRC in humans and rats, with limited research on CYP2C subtypes in mice, our study indicates a potential role for CYP2C38 in mice (29, 30). Consistent with studies on VRC metabolism in other species (20, 31), our results show that LPS-induced inflammation in mice decreased VRC metabolism, accompanied by elevated proinflammatory factors and reduced IL-10 expression. However, 1-MNA administration reversed this inflammatory response, improving both IL-10 secretion and CYP2C38 gene expression. These findings corroborate previous clinical research suggesting that inflammatory conditions can impair VRC metabolism (32). Moreover, our study supports reports indicating that LPS-induced inflammation suppresses CYP2C38 expression, leading to a reduction in VRC metabolism.

Further investigation into the effects of 1-MNA administration alone revealed minimal impact on VRC metabolism. However, in LPS-induced inflammatory conditions, 1-MNA significantly upregulated drug-metabolizing enzymes, indicating a potential indirect regulatory role in enhancing CYP2C38 expression to facilitate VRC metabolism (**Figures 1C–O**). 1-MNA administration resulted in a significant increase in IL-10 expression, warranting further investigation into its role. Previous studies have shown that KCs are susceptible to polarization, which affects hepatocyte inflammation (33, 34). In this study, isolated KCs undergoing M2 polarization exhibited increased IL-10 expression. In addition, a significant negative correlation was found between the M1/M2 ratio and IL-10 levels (**Figure 2F**). These findings suggest that KCs play a crucial role in facilitating IL-10 secretion, which may enhance the capacity of hepatocytes to metabolize VRC.

Our preliminary investigation indicated the potential involvement of 1-MNA and IL-10 in VRC metabolism. Specifically, 1-MNA did not significantly impact the expression of VRC drug-metabolizing enzymes in hepatocytes (**Figure 3E**), whereas IL-10 administration significantly increased the expressions of CYP2C38 and CYP2C29 (**Figure 3F**). This suggests that IL-10 may stimulate the expression of CYP2C isoforms in hepatocytes, representing a significant discovery. Building on this observation, we referenced established research methodologies (20, 35) to further explore the mechanism by which IL-10 regulates CYP2C38 expression in hepatocytes under inflammatory conditions.

Primary hepatocytes are the functional cells responsible for drug metabolism, and understanding the stimulatory effects of the external environment on these cells is crucial for elucidating the mechanisms by which the environment influences drug metabolism. NF-κB plays a pivotal role in regulating both the circadian clock and drug-metabolizing enzymes (8). In this study, we observed that IL-10 suppresses the transcriptional activity of p65 within the nuclei of hepatocytes. ChIP assays targeting p65 and PXR further confirmed that IL-10 promotes the transcriptional upregulation of PXR by inhibiting nuclear p65 expression (**Figure 4E**). These findings align with previous research that demonstrated the inhibitory effect of nuclear p65 on the transcriptional expression of PXR (36). In addition, exogenous compounds can reduce CYP3A4 enzyme activity by inhibiting the effect of NF-κB on PXR (37).

The results from the metabolic activity assays in IL-10 gene knockout mice also support the hypothesis that IL-10 enhances VRC metabolism and reduces its accumulation in hepatocytes. This occurs through the inhibition of the effect of p65 on PXR and the subsequent upregulation of CYP2C38 activity (**Figure 5D**). We identified CYP2C38 as a critical enzyme for promoting VRC metabolism in response to 1-MNA and IL-10. However, further experiments are necessary to confirm whether the metabolic functions of CYP2C38 in mouse hepatocytes align with those of the human VRC-metabolizing enzyme CYP2C19. Such verification would strengthen the scientific validity of the role of IL-10 in enhancing VRC metabolism and support its potential clinical applications.

Moreover, we primarily focused on the involvement of the NF-κB/PXR signaling pathway in VRC metabolism. Previous research has highlighted the role of the PXR/NF-κB pathway in modulating inflammatory responses and influencing the transcriptional regulation of metabolic enzymes in hepatocytes (38–40). Although our results indicate a significant role of this pathway in the expression and function of CYP2C38, they did not exclude the possibility of additional impacts of the PXR/NF-κB signaling pathway on other aspects of drug metabolism. Thus, further investigation into the complex interplay between NF-κB and PXR in hepatocytes, particularly following IL-10 stimulation, is necessary to fully understand their collective influence on drug-metabolizing enzymes such as CYP2C38.

## 5 Conclusions

**Figure 6:**
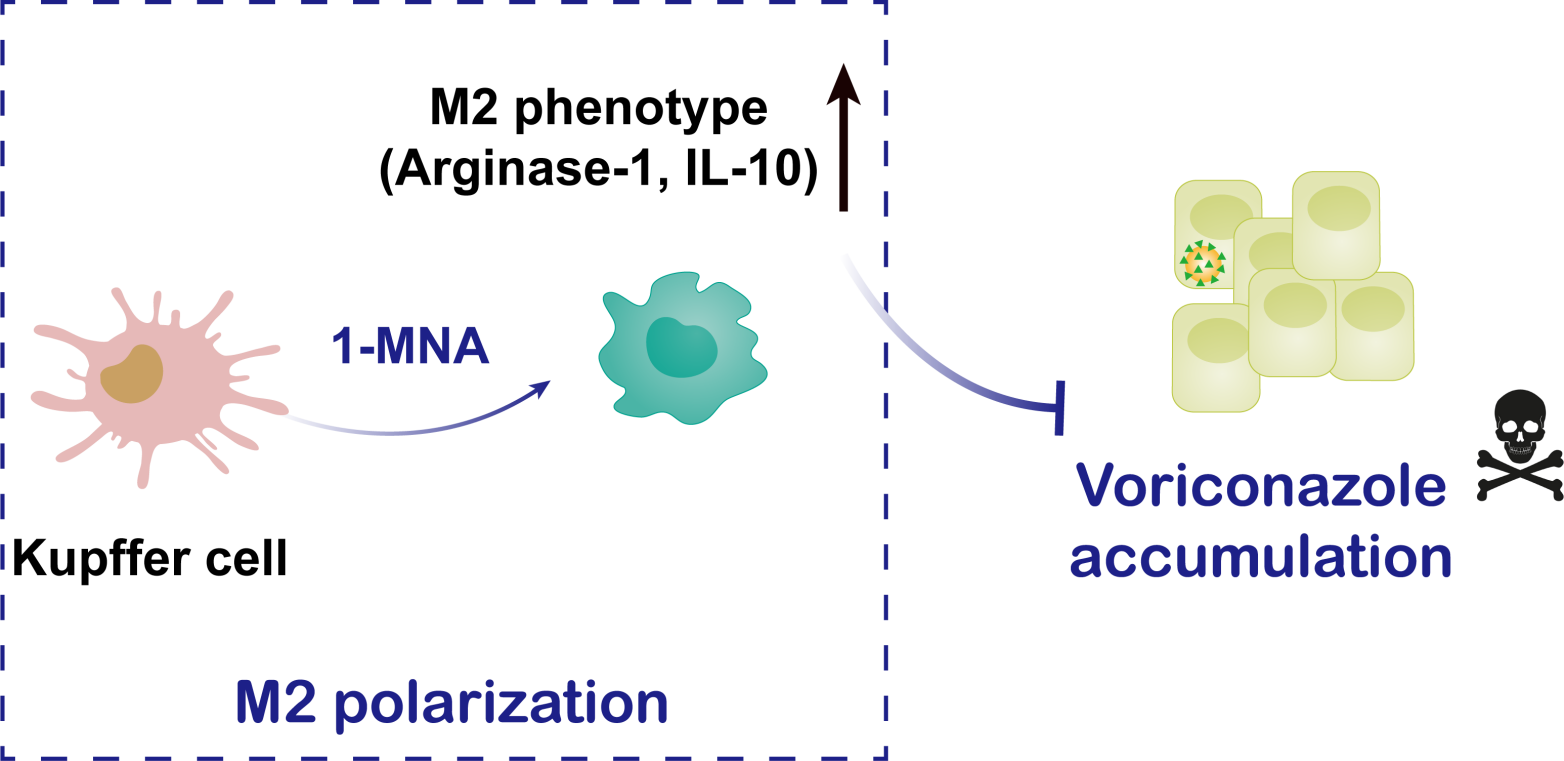
Schematic illustration of 1-MNA regulatory mechanism of VRC metabolism.

This study highlights the potential of 1-MNA in inhibiting VRC accumulation under inflammatory conditions by modulating the M2 polarization of KCs in the liver, stimulating IL-10 secretion, and alleviating the inhibitory effects of NF-κB-p65 on CYP2C38 expression in hepatocytes (**Figure 6**). Through these mechanisms, 1-MNA facilitates the metabolism of VRC, offering new insights into how the inflammatory environment can be manipulated to improve drug metabolism and reduce adverse drug reactions in clinical settings.

## Data Availability

The original contributions presented in the study are included in the article/**Supplementary Material**. Further inquiries can be directed to the corresponding author/s.

## References

[1] Walsh TJ Anaissie EJ Denning DW Herbrecht R Kontoyiannis DP Marr KA. Treatment of aspergillosis: clinical practice guidelines of the Infectious Diseases Society of America. Clin Infect Dis. (2008) 46:327–60. doi: 10.1086/525258 18177225

[2] Salmanton-García J Au W-Y Hoenigl M Chai LYA Badali H Basher A. The current state of laboratory mycology in Asia/Pacific: A survey from the European Confederation of Medical Mycology (ECMM) and International Society for Human and Animal Mycology (ISHAM). Int J Antimicrob Agents. (2023) 61:106718. doi: 10.1016/j.ijantimicag.2023.106718 36640851

[3] Wang X Tong Y Xun T Feng H Lei Y Li Y. Functions, mechanisms, and therapeutic implications of noncoding RNA in acute myeloid leukemia. Fundam Res. (2023) 5:1–15. doi: 10.1016/j.fmre.2023.04.012 PMC12327837 40777810

[4] Wang T Miao L Shao H Wei X Yan M Zuo X. Voriconazole therapeutic drug monitoring and hepatotoxicity in critically ill patients: A nationwide multi-centre retrospective study. Int J Antimicrob Agents. (2022) 60:106692. doi: 10.1016/j.ijantimicag.2022.106692 36372345

[5] Li H Li M Yan J Gao L Zhou L Wang Y. Voriconazole therapeutic drug monitoring in critically ill patients improves efficacy and safety of antifungal therapy. Basic Clin Pharmacol Toxicol. (2020) 127:495–504. doi: 10.1111/bcpt.13465 32639669

[6] Wang X Zhao J Wen T Liao X Luo B. Predictive value of FMO3 variants on plasma disposition and adverse reactions of oral voriconazole in febrile neutropenia. Pharmacology. (2021) 106:202–10. doi: 10.1159/000510327 32998136

[7] Wang X Ye C Xun T Mo L Tong Y Ni W. Bacteroides fragilis polysaccharide A ameliorates abnormal voriconazole metabolism accompanied with the inhibition of TLR4/NF-κB pathway. Front In Pharmacol. (2021) 12:663325. doi: 10.3389/fphar.2021.663325 PMC8115215 33995087

[8] Wang X Rao J Tan Z Xun T Zhao J Yang X. Inflammatory signaling on cytochrome P450-mediated drug metabolism in hepatocytes. Front In Pharmacol. (2022) 13:1043836. doi: 10.3389/fphar.2022.1043836 PMC9637984 36353494

[9] Wang P Liu S Yang J. Physiologically based pharmacokinetic modeling to investigate the disease-drug-drug interactions between voriconazole and nirmatrelvir/ritonavir in COVID-19 patients with CYP2C19 phenotypes. Clin Pharmacol Ther. (2024) 116:363–371. doi: 10.1002/cpt.3222 38429919

[10] Liu S Yao X Tao J Zhao S Sun S Wang S. Impact of CYP2C19, CYP2C9, CYP3A4, and FMO3 genetic polymorphisms and sex on the pharmacokinetics of voriconazole after single and multiple doses in healthy chinese subjects. J Clin Pharmacol. (2024) 64:1030–1043. doi: 10.1002/jcph.2440 38654529

[11] Hinze CA Fuge J Grote-Koska D Brand K Slevogt H Cornberg M. Factors influencing voriconazole plasma level in intensive care patients. JAC Antimicrob Resist. (2024) 6:dlae045. doi: 10.1093/jacamr/dlae045 38500519 PMC10946233

[12] Rao J Qiu P Zhang Y Wang X. Gut microbiota trigger host liver immune responses that affect drug-metabolising enzymes. Front Immunol. (2024) 15:1511229. doi: 10.3389/fimmu.2024.1511229 39720713 PMC11668346

[13] Schmeisser K Mansfeld J Kuhlow D Weimer S Priebe S Heiland I. Role of sirtuins in lifespan regulation is linked to methylation of nicotinamide. Nat Chem Biol. (2013) 9:693–700. doi: 10.1038/nchembio.1352 24077178 PMC4076143

[14] Sidor K Jeznach A Hoser G Skirecki T. 1-Methylnicotinamide (1-MNA) inhibits the activation of the NLRP3 inflammasome in human macrophages. Int Immunopharmacol. (2023) 121:110445. doi: 10.1016/j.intimp.2023.110445 37290319

[15] Roca Suarez AA Plissonnier M-L Grand X Michelet M Giraud G Saez-Palma M. TLR8 agonist selgantolimod regulates Kupffer cell differentiation status and impairs HBV entry into hepatocytes via an IL-6-dependent mechanism. Gut. (2024) 73:2012–2022. doi: 10.1136/gutjnl-2023-331396 PMC12210347 38697771

[16] Yao J-M Ying H-Z Zhang H-H Qiu F-S Wu J-Q Yu C-H. Exosomal RBP4 potentiated hepatic lipid accumulation and inflammation in high-fat-diet-fed mice by promoting M1 polarization of Kupffer cells. Free Radic Biol Med. (2023) 195:58–73. doi: 10.1016/j.freeradbiomed.2022.12.085 36572267

[17] Wang Q Wei S Zhou H Shen G Gan X Zhou S. Hyperglycemia exacerbates acetaminophen-induced acute liver injury by promoting liver-resident macrophage proinflammatory response via AMPK/PI3K/AKT-mediated oxidative stress. Cell Death Discovery. (2019) 5:119. doi: 10.1038/s41420-019-0198-y 31341645 PMC6642179

[18] López-Navarrete G Ramos-Martínez E Suárez-Álvarez K Aguirre-García J Ledezma-Soto Y León-Cabrera S. Th2-associated alternative Kupffer cell activation promotes liver fibrosis without inducing local inflammation. Int J Biol Sci. (2011) 7:1273–86. doi: 10.7150/ijbs.7.1273 PMC3221364 22110380

[19] Rajak S. Dynamics of cellular plasticity in non-alcoholic steatohepatitis (NASH). Biochim Biophys Acta Mol Basis Dis. (2024) 1870:167102. doi: 10.1016/j.bbadis.2024.167102 38422712

[20] Wang X Hu X Ye C Zhao J Tan SC Zhou L. Astragalus Polysaccharide Enhances Voriconazole Metabolism under Inflammatory Conditions through the Gut Microbiota. J Clin Trans Hepatology. (2024) 12:481–95. doi: 10.14218/JCTH.2024.00024 PMC11106349 38779521

[21] Zhang W Liu K Ren G-M Wang Y Wang T Liu X. BRISC is required for optimal activation of NF-κB in Kupffer cells induced by LPS and contributes to acute liver injury. Cell Death Disease. (2023) 14:743. doi: 10.1038/s41419-023-06268-z 37968261 PMC10651896

[22] Taniki N Nakamoto N Chu P-S Mikami Y Amiya T Teratani T. Intestinal barrier regulates immune responses in the liver via IL-10-producing macrophages. JCI Insight. (2018) 3:e9198. doi: 10.1172/jci.insight.91980 PMC6124432 29925685

[23] Yu H Xu H Yang X Zhang Z Hu J Lu J. Gut microbiota-based pharmacokinetic-pharmacodynamic study and molecular mechanism of specnuezhenide in the treatment of colorectal cancer targeting carboxylesterase. J Pharm Anal. (2023) 13:1024–40. doi: 10.1016/j.jpha.2023.06.012 PMC10568112 37842660

[24] Sun D Yang Y-M Jiang H Wu H Ojaimi C Kaley G. Roles of CYP2C29 and RXR gamma in vascular EET synthesis of female mice. Am J Physiol Regul Integr Comp Physiol. (2010) 298:R862–9. doi: 10.1152/ajpregu.00575.2009 PMC2853397 20130225

[25] Löfgren S Baldwin RM Carlerös M Terelius Y Fransson-Steen R Mwinyi J. Regulation of human CYP2C18 and CYP2C19 in transgenic mice: influence of castration, testosterone, and growth hormone. Drug Metab Dispos. (2009) 37:1505–12. doi: 10.1124/dmd.109.026963 PMC2698944 19339376

[26] Xu X-S Feng Z-H Cao D Wu H Wang M-H Li J-Z. SCARF1 promotes M2 polarization of Kupffer cells via calcium-dependent PI3K-AKT-STAT3 signalling to improve liver transplantation. Cell Prolif. (2021) 54:e13022. doi: 10.1111/cpr.13022 33686740 PMC8016636

[27] Wei K Zhang H Yang S Cui Y Zhang B Liu J. Chemo-drugs in cell microparticles reset antitumor activity of macrophages by activating lysosomal P450 and nuclear hnRNPA2B1. Signal Transduct Target Ther. (2023) 8:22. doi: 10.1038/s41392-022-01212-7 36658134 PMC9852455

[28] Wang S Lin Y Li F Qin Z Zhou Z Gao L. An NF-κB-driven lncRNA orchestrates colitis and circadian clock. Sci Adv. (2020) 6:eabb5202. doi: 10.1126/sciadv.abb5202 33055157 PMC7556837

[29] Zubiaur P Soria-Chacartegui P Boone EC Prasad B Dinh J Wang WY. Impact of CYP2C:TG haplotype on CYP2C19 substrates clearance *in vivo*, protein content, and *in vitro* activity. Clin Pharmacol Ther. (2023) 114:1033–42. doi: 10.1002/cpt.3012 PMC10592245 37528442

[30] Muhareb A Blank A Meid AD Foerster KI Stoll F Burhenne J. CYP3A and CYP2C19 activity determined by microdosed probe drugs accurately predict voriconazole clearance in healthy adults. Clin Pharmacokinet. (2023) 62:1305–14. doi: 10.1007/s40262-023-01287-7 PMC10450012 37505445

[31] Wang T Zhu H Sun J Cheng X Xie J Dong H. Efficacy and safety of voriconazole and CYP2C19 polymorphism for optimised dosage regimens in patients with invasive fungal infections. Int J Antimicrob Agents. (2014) 44:436–42. doi: 10.1016/j.ijantimicag.2014.07.013 25239277

[32] Cheng L Xiang R Liu F Li Y Chen H Yao P. Therapeutic drug monitoring and safety of voriconazole in elderly patients. Int Immunopharmacol. (2020) 78:106078. doi: 10.1016/j.intimp.2019.106078 31830620

[33] Yu Y Liu Y An W Song J Zhang Y Zhao X. STING-mediated inflammation in Kupffer cells contributes to progression of nonalcoholic steatohepatitis. J Clin Invest. (2019) 129:546–55. doi: 10.1172/JCI121842 PMC6355218 30561388

[34] Dong B Zhou Y Wang W Scott J Kim K Sun Z. Vitamin D receptor activation in liver macrophages ameliorates hepatic inflammation, steatosis, and insulin resistance in mice. Hepatol (Baltimore Md). (2020) 71:1559–74. doi: 10.1002/hep.30937 31506976

[35] Ma L Zeng W Tan Z Wang R Yang Y Lin S. Activated hepatic nuclear factor-κB in experimental colitis regulates CYP2A5 and metronidazole disposition. Mol Pharm. (2023) 20:1222–9. doi: 10.1021/acs.molpharmaceut.2c00890 36583631

[36] Gu X Ke S Liu D Sheng T Thomas PE Rabson AB. Role of NF-kappaB in regulation of PXR-mediated gene expression: a mechanism for the suppression of cytochrome P-450 3A4 by proinflammatory agents. J Biol Chem. (2006) 281:17882–9. doi: 10.1074/jbc.M601302200 16608838

[37] Li Y Lin N Ji X Mai J Li Q. Organotin compound DBDCT induces CYP3A suppression through NF-κB-mediated repression of PXR activity. Metallomics. (2019) 11:936–48. doi: 10.1039/c8mt00361k 30848264

[38] Zhang J Cao L Wang H Cheng X Wang L Zhu L. Ginsenosides regulate PXR/NF-κB signaling and attenuate dextran sulfate sodium-induced colitis. Drug Metab Dispos. (2015) 43:1181–9. doi: 10.1124/dmd.115.063800 25986850

[39] Liu M Zhang G Zheng C Song M Liu F Huang X. Activating the pregnane X receptor by imperatorin attenuates dextran sulphate sodium-induced colitis in mice. Br J Pharmacol. (2018) 175:3563–80. doi: 10.1111/bph.14424 PMC6086988 29945292

[40] Li F Wang X Cai Y Lin Y Tang Y Wang S. Gut microbiota-derived metabolites as novel therapies for inflammatory bowel diseases: Role of nuclear receptors. Fundam Res. (2024) 3:1–8. doi: 10.1016/j.fmre.2024.01.018 PMC12744613 41467030

