# Supplementary material for: 1-methylnicotinamide modulates IL-10 secretion and voriconazole metabolism
Source: Front Immunol. 2025 Feb 13;16:1529660. doi: 10.3389/fimmu.2025.1529660 (PMC11865947; doi:10.3389/fimmu.2025.1529660)
Supplement: Supplementary file 1 [file Table1.docx]

**Supplementary Table 1.** **Primers used in the study**

| **GENE** | **PRIMER** | **PRIMER SEQUENCE** |
| --- | --- | --- |
| qRT-PCR | | |
| Mouse *Cyp2C38* | FW^a^ | GGTCAGAAGTCCTTCATCGCCA |
|  | RV^b^ | TGAAGCTGCTCTCAGGATTCCC |
| Mouse *Cyp2c29* | FW | GCTCTCCTACTCCTGCTGAAGT |
|  | RV | ATGTGGCTCCTGTCTTGCATGC |
| Mouse *Cyp3a11* | FW | ACAGCACTGGTCAGAGCCTGAA |
|  | RV | GAGAGCAAACCTCATGCCAAGG |
| Mouse *P-gp* | FW | GGACACCATTGAGAAGCTGAAGG |
|  | RV | GTCTTGAGTCCTCGCTGTGTGA |
| Mouse *PXR* | FW | CATCTCAGCAACCCACACAG |
|  | RV | GGGGTCATAGGAGTCATTGG |
| Mouse *CAR* | FW | CTCAAGGAAAGCAGGGTCAGC |
|  | RV | GGATCCACCATGGCCAGTAGGGAAGATGAG |
| Mouse *AhR* | FW | AGCCGGTGCAGAAAACAGTAA |
|  | RV | AGGCGGTCTAACTCTGTGTGT |
| Mouse *GAPDH* | FW | CTGAGTACGTCGTGGAGTC |
|  | RV | AAATGAGCCCCAGCCTTC |
| ChIP | | |
| Mouse *PXR-BR^c^* | FW | TGCACACAGGTTCCTGTTCCTGA |
|  | RV | GGGGTGCGTGTCCTGGATGC |
| Mouse *PXR-NR^d^* | FW | TCCGGAAAGATCTGTGCTCT |
|  | RV | AGGGAGATCTGGTCCTCGAT |

^a^ FW, Forward; ^b^ Reverse; ^c^ BR, binding region; ^d^ NR, non-binding region.

**Supplementary Table 2. Fluorescent antibodies**

| **ANTIBODIES** | **SOURCE** | **IDENTIFIER** |
| --- | --- | --- |
| FITC anti-mouse F4/80 | Biolegend | 123108, BM8 |
| APC anti-mouse F4/80 | Biolegend | 123116, BM8 |
| PE anti-mouse iNOS | Biolegend | 696806, W16030C |
| FITC anti-mouse CD206 | Biolegend | 141704, C068C2 |
